# Barriers and facilitators to contraceptive use among Somali immigrant women in Oslo: A qualitative study

**DOI:** 10.1371/journal.pone.0229916

**Published:** 2020-03-10

**Authors:** Abdi A. Gele, Fathia K. Musse, Mary Shrestha, Samera Qureshi

**Affiliations:** 1 Department of Health Service Research, Norwegian Institute of Public Health, Oslo, Norway; 2 Department of Family Counselling, Oslo Municipality, Oslo, Norway; Kwame Nkrumah University of Science and Technology, GHANA

## Abstract

**Background:**

The European Action Plan for Sexual and Reproductive Health emphasizes the importance of improving access to contraceptive services for disadvantaged groups. However, a prior study showed that the prevalence of abortion is two times higher among refugees compared to non-immigrants in Norway. Similarly, a recent study reported that 50% of Somali women in Oslo had unintended childbirth on one occasion or more. These findings are supported by several studies in Europe that showed immigrant and refugee women have higher rates of unintended pregnancy and abortion than Non-immigrant women, and more than half of immigrants, who seek abortion are not using any form of contraception, raising concerns about their access to utilization of modern contraception. However, none of these studies have explored reasons underlying immigrant women’s underutilization of modern contraception. The present study aimed to explore the barriers and facilitators to contraceptive usage among Somali immigrant women in Oslo area.

**Methods:**

A qualitative study using unstructured in-depth interviews with twenty one Somali women of reproductive age, >18 years, was conducted in Oslo from May—August 2018. The participants were recruited using purposive sampling method. Interviews began with a general question and were followed with some probing questions, and were continued until data saturation was reached. Data were analyzed using thematic analysis.

**Results:**

Although the majority of the participants were educated, aware of the importance of contraceptive methods and interested in child spacing, systemic and socio-cultural barriers were found to be hindering their access to contraception. Several barriers were identified, including: language problems, lack of adequate information, religious beliefs, gender roles and social pressure.

**Conclusion:**

Eliminating the barriers which prevent women from receiving their desired form of contraception will have important public health implications, including lengthening inter-pregnancy intervals, and fewer unplanned pregnancies and abortions. These findings can support policy makers, civil society organizations and health providers to develop cultural sensitive programmes and educational interventions, which help Somali immigrant women overcome the identified barriers to contraception.

## Background

Ensuring access to modern contraceptive methods for women is a key to securing not only the health and well-being of women, but also the health and development of communities [1]. The World Health Organization (WHO) have recognized that tailored reproductive services are not only critical to improving the health of women, but also a human right. Currently, about 214 million women have an unmet need for modern contraceptives [2]. Over 85 million mistimed or unwanted pregnancies occur each year worldwide, which leads to high rates of induced abortions and maternal morbidity and mortality [3]. The European Action Plan for Sexual and Reproductive Health emphasizes the importance of improving access to contraceptive services of disadvantaged groups, such as immigrants [4]. Nonetheless, studies from Europe suggest that immigrant and refugee women have higher rates of unintended pregnancy and abortion than non-immigrant women [5–9], raising concerns about their access to quality contraceptive education and healthcare [10, 11]. Unfair differences in access to reproductive health services within and between groups are defined by the World Health Organization (WHO) as inequity [7].

Research undertaken in Scandinavia suggests that immigrant women seek induced abortion in higher numbers than would be expected given their proportion of the population [8, 11, 12]. A registry-based study in Norway found a pregnancy termination rate of 30.2 per 1000 women per year among refugees compared with a 16.7 per 1000 women per year rate among non-migrants [10]. Another study in Norway found that 25% of women seeking abortion were immigrants from non-Western countries [11]. Immigrant women are vulnerable to unintended pregnancies owing to their limited knowledge about and access to services for contraception and family planning [8, 12, 13]. Furthermore, the underutilization of contraceptives does not only result in unintended pregnancies and abortion, it also restricts women’s ability to achieve educational and economic goals [14]. Reproductive rights include the right of women to decide how many children they want to have, full access to family planning information and services [15]. Facilitating access to contraception for women has the potential benefit of improving maternal health [16] by lowering the annual number of unintended pregnancies.

The Norwegian healthcare system is predominantly public and everyone is entitled to have a general practitioner (GP) who can prescribe contraceptives and insert implants and intrauterine devices (IUDs). There is co-payment for the GP visit, while midwives and nurses can also prescribe contraceptives. Unlike GPs, the visits to midwives and nurses are free of charge. Contraceptive methods are subsidized partly or wholly for women under the age of 22 while older women must pay the full cost of the method. The utilization of reproductive services depends on language proficiency, and comprehension of information received about the health system. Doctor-patient interaction patterns, and language and cultural differences between immigrants and health providers are critical in immigrant’s access to services [17].

An estimated 43,000 Somali immigrants live in Norway, thus constituting the largest non-Western immigrants in the country. Somalia is an East-African country with a population of 12 million and it has the lowest contraceptive prevalence rate in the world of <10% [18]. Understanding and utilization of modern contraceptives is low amongst Somali women, including those who desire to avert pregnancy [19]. Somali women’s fertility rate is 6.4, which exceeds fertility rate in sub-Saharan Africa (5.1) and the world (2.5). This is partly reflected in the high maternal mortality rate of Somalia (732 per 100,000), which is much high than the average in sub-Saharan Africa (84.5 per 100,000) [20]. Total Fertility rate of Somali women in Norway is 4.40 [21], which is much higher than the total fertility rate in Norway of 1.78. Religious misperceptions, cultural factors, and a lack of knowledge regarding modern contraceptives prevent both Somali women and men from accepting and accessing contraceptive health services [22]. Large families are valued in Somali culture, and therefore there is a social pressure for women to bear more children [23].

Somali immigrants may bring their beliefs and attitudes toward contraception with them during migration. For instance, the main reasons for Somali immigrant women in Finland not using contraception were reported to be religious objections and issues related to gender roles [24]. This mirrors the rationale of women in Somalia, where using contraceptives with the intention to limit the number of children is considered to be against Islamic values and Somali traditions [22], further highlighting that the culture of immigrant’s country of origin may shape their contraceptive use regardless of availability of the methods. Nevertheless, cultural traditions, religious misperceptions and social norms are not static but, rather, a dynamic process that changes with the circumstances that surround it [25]. Migration to a new country, exposure to different social context, and improved educational levels can influence traditions and cultural beliefs of individuals, which in turn play a key role in reshaping a community’s norms, behaviors, and values [25].

A number of studies in Norway reported a low utilization of modern contraception among immigrant women [10, 11, 26]. However, none of these studies have explored reasons underlying immigrant women’s underutilization of modern contraception. This study thus, provides the first qualitative study on the barriers and facilitators to modern contraception among Somali immigrant women in Norway.

## Methods

### Sample and sampling technique

A qualitative study using unstructured in-depth interviews was conducted in Oslo from May to August 2018. Oslo is the capital of Norway, and almost 50% of Somali immigrants in Norway live in the Oslo area. We have chosen unstructured in-depth interviews for this study, because it is not only a flexible tool for exploring people’s experiences and their attitudes of reality, but it is also a tool that does not impose *a priori* categorization of the questions, which may limit the field of inquiry [33]. Twenty-one Somali women, aged >18; all of them were first-generation immigrants were recruited using purposive sampling technique. Participants were recruited from different social settings and they were selected based on their knowledge on the topic and their connection to Somali community in Oslo area. Norwegian Regional Committee for medical and health research ethics approved the study with approval number: 2017/2386. As some of the participants were illiterate, oral consent were obtained from all participants.

### Data collection

The participants were interviewed one on one in Somali language, either at their homes or at a selected place of their choice. Data were collected using an interview guide but questions were asked to women in a convenient way, and the order of the questions varied from interview to interview. The interview started with open-ended questions to collect participants’ demographic characteristics as shown in Table 1. The participants were offered the opportunity to share detailed information of their experiences and opinions about contraception utilization of Somali women [27, 28]. The unstructured interview technique allowed women to go beyond the actual questions and to talk freely about the contraception methods to highlight additional issues of concern, which allowed for the exploration of unanticipated themes. Thus, the participants presented not only their personal experiences but also the experience of their acquaintances and friends. The interview guide covered several themes: (1) community norms and communication regarding family planning (2); knowledge and attitudes toward modern contraceptives (3); reasons for usage or non-usage of modern contraceptives; and (4) factors that prevent Somali women from utilizing modern contraception. Lastly, the acceptable means of increasing access and utilization of contraception were explored through women’s perspectives. The interviews lasted for 45 to 60 minutes. All the interviews were audio-recorded with consent. The second author, who is a Somali ethnic female with extensive experience in qualitative data collection, carried out the interviews, together with the first author. Recruitment and interviews continued until researchers became convinced that no new information was emerging from the additional interviews, that is, when saturation was achieved.

**Table 1:** Characteristics of study participants.

No.	Age	Employment	Years of living in Norway	No. of children
1	37	Employed	15	2
2	28	Job training	11	4
3	34	Job training	10	4
4	42	Employed	28	1
5	35	Employed	19	5
6	46	Employed	26	2
7	44	Unemployed	26	1
8	43	Employed	28	3
9	39	Employed	28	3
10	25	Job training	10	2
11	33	Job training	7	9
12	34	Employed	25	2
13	28	Job training	7	2
14	26	Employed	24	2
15	28	Employed	14	2
16	29	Student	18	3
17	32	Job training	10	4
18	37	Job training	19	2
19	44	Job training	17	10
20	46	Job training	26	6
21	37	Job training	7	4

### Analysis

The second author transcribed the interviews verbatim. The transcripts were translated into English for coding, and were carefully read several times by the first and the third authors for accuracy and completeness. Thematic analysis was used to identify and analyze important themes [29]. Themes were identified by bringing together fragments of stories, experiences, and beliefs that are often meaningless when viewed separately [30]. Afterwards, themes that emerged from the women's stories were pieced together to form a comprehensive picture of the participants' shared experience. The themes were divided into categories based on the participants' experience in barriers and facilitators to contraception use. In our previous paper, we presented that nearly 50% of Somali women in Oslo had unintended childbirth on one or more occasions [31], which supports the qualitative findings that are presented in this paper. Thus, the consistency of the findings from the two methods (qualitative and quantitative data) have served to ensure the trustworthiness and credibility of the study's results. Furthermore, three study participants who could speak English have read the final paper and verified that what they said is presented correctly.

## Results

As shown in Table 1, majority of study participants have been living in Norway for an average (± standard deviation) of 17.9 ± 7.8 years. After the analysis, four main categories emerged from the data. Two of these categories represented barriers to contraception use, while the other two have represented facilitators to contraception use. These categories were summarized in Table 2.

**Table 2:** Themes and subthemes.

Theme	Subthemes
**Barriers**	
**System related barriers**	Language
	Lack of information
	Perceived side-effects of contraception
	Cost
**Socio-cultural barriers**	Religion
	Social pressure
	Husband against contraception use
**Facilitators**	
**System based facilitators**	Information in native language
	Increasing multicultural communication skills of health providers
	Religious leaders as partners in contraception use
**Community based facilitators**	Learning Norwegian language
	Empowerment of women

## System related barriers

In this paper, system related barriers refer to users’ inability to access relevant contraceptive information and services mainly due to system related factors.

### Lack of Norwegian language skills

Poor knowledge of Norwegian language was identified as a barrier in accessing contraception. One participant narrated her experience with unintended\mistimed pregnancies due to lack of knowledge about the system and the language. Eventually, she decided to use her husband as an interpreter, but the husband was not in favor of contraception. She believes that he concealed the information about contraception from her. This has resulted in unintended pregnancies that could be averted if the women could speak the Norwegian language or if she had known about her rights to a professional interpreter.

*I did not receive information about contraception when I needed it most*. *I have got children that are very closely spaced*. *I wanted to space them with three years of interval but I never had the correct information of how to do it*. *I did not know about the existence of modern contraception*. *I was new to the country and I was at home all the time*. *I was relying on an information given to me by an interpreter (husband)*. *Maybe he was told about the importance of contraception but he never told me*. *I received the information too late after having many children but it was still good to have the information, even though it was late (46 years old in job training)*.

Another participant reported that many Somali women are reluctant to seek information about contraception to their GPs, because they do not speak Norwegian language. They have little trust to health providers. For that, they rely on information they are given by other women, friends and acquaintances.

*Women who do not understand the Norwegian language, have fears and doubts about the information they are given by their GPs*, *instead, they prefer to seek information from people they can communicate with in their native language (44 years old in job training)*.

### Lack of adequate information

Lack of adequate information about contraception is among the widely mentioned barriers to access. Majority of study participants reported that Somali women receive information about contraception when they already have many, closely spaced, children. Some women were reported to have enough information, yet they are against the use of contraception for unaddressed concerns.

*Somali women start using contraception when they already have many children up to 6 or more*. *Many people are new to the country, and they might not have received any information about contraception*. *(46 years old in job training)*.

In the absence of appropriate health information about contraception, women receive misleading information from other women, and instead of seeking information from health providers, they are stuck in myths and misperceptions from lay peers, which enforce their doubts and fears of modern contraception.

*I have never used contraception*. *The reason was that my friends used it and they experienced many health problems, which motivated me to stop using it*. *My friends think that contraception is dangerous to health*. *The women I know do not like contraception (25 years old in job training)*.

### Perceived side-effects of contraception

Fear about side-effects of contraception such as bleeding, irregular menstruation, cancer and vitamin deficiencies were reported by vast majority of the study participants. In addition, participants reported widespread rumors they hear in their community such as; many Somali women who used contraception and who eventually become infertile, or who have developed dangerous illnesses such as cancer.

*If you use contraception for long time, it can reduce the quality of your eggs*. *Therefore, you may not become pregnant after having used contraception for a long time. (42 years old, employed)*

*It causes vitamin deficiencies, excessive bleeding*, *and that motivated me not to use contraception (25 years old in job training)*.

### High cost of contraceptives

According to some participants, high cost of the modern contraception was reported to be a barrier to contraceptive use. Most Somali women do not have paid jobs. Consequently, they may be unable to buy modern contraception, as they have to use their limited income to meet other more important family needs.

*In Norway, contraception is available, but it is not cheap; it is available for those who can afford to buy it*. *I think some women can afford it, but others cannot, depending on their financial situation*. *(42 years old, employed)*.

*Contraception is, in fact, expensive. I bought it 2500 (Norwegian kroner) …*.. *Hormonal contraception are expensive to people who do not work (28 years old, in job training)*.

## Socio-cultural barriers

Socio-cultural barriers refer to factors that impede women’s utilization of modern contraceptives for religious believes, social norms, social pressure, husband not in favor of using it and so on.

### Religious beliefs not favoring contraceptive use

Some participants believed that their community looks unfavorably upon the use of contraception for religious reasons. Many participants reported that they have asked religious leaders about the use of contraception and they were told not to use contraception but to respect the natural order established by God. According to other participants, the issue of contraception use is a controversial issue among religious leaders, with some rejecting while others accepting the use of contraception. Given the controversial nature of the contraception among religious leaders, the ultimate decision of who to listen and whether to use contraception is left for the women.

*A religious leader told me that it is not allowed to take contraception*. *There are some religious leaders who believe that it is allowed but most of them think it is not allowed*. *Most known Somali sheikhs like xxx said that contraception is not allowed. (33 years old in job training)*

However, some participants were not in favor of this religious view. They presented examples of many Somali families who have problems coping with the needed care of their children. One participant, who was knowledgeable and has faith about Islam has reasoned why women should use contraception. She believes that women will not be punished by Allah for not having children, but Allah will punish for those who do not provide the necessary care for their children. This means that the Islam supports for women to have children that they can manage.

*If you have many children that you cannot manage their lives, Allah may punish you for not giving good care to your children*. *But if you do not become pregnant and not have a single child, Allah will not punish you for not having children (46 years old in job training)*.

### Social pressure to have large family

Cultural norms that support large family size were reported to be upheld by many Somali families in Norway, which serve as deterrent on the use of contraception. According to the participants, women are influenced by the peer pressure to have a large family, and the primary pressure mostly comes from other women and mothers. The peers often have several children, and they intercede when they see that a woman has few children by advising her to have more.

*The reason why many women do not use contraception is mainly because of the influence of peers who maintain the social norm of having many children*. *Women around you will put pressure on you, which pushes you to have many children like them. (46 years old, unemployed)*.

*When everyone around you have many children, there is a pressure on you to have many children like them*. *I have two children, and people often ask me why I do not have many children early, as my reproductive age is limited and I will soon reach menopause. (46 years old, employed)*.

### Husband’s resistance to contraception

Some participants pointed out that the husbands’ resistance to contraception is a major barrier for women’s contraception use. According to some participants, the husband’s support for contraceptive use is a decisive factor for women’s contraception use. Participants also mentioned that husbands’ desire for large family size and their attitude against contraception use, have prompted many Somali women to break up their marriage. All participants agreed that couples do not discuss the number of children that would be best for their family.

*My husband was against contraception*. *He was very much against; he always wanted more children and he was also against spacing or stopping pregnancies*. *Finally, I decided to leave my husband and ask for divorce. (35 years old, employed)*.

*My husband was not happy that I should use contraception*. *I did not inform him that I am going to use contraception because I knew he is against it*. *He came from a family with many children (46 years old, unemployed)*.

*Some women believe that the husband will abandon them if they do not give birth many children*. *(46 years old, employed)*.

## System based facilitators

### Information in native language

Participants expressed that if the information were given to them in their native language at health centers, it would help increase the contraception uptake. Women who receive information in their own language understand the importance of contraception, and enable them to openly discuss their concerns and fears surrounding contraceptive methods with health professionals. This may clear away all myths and misperceptions surrounding contraception.

*Health stations and health centers should employ people who can communicate with women in their own language*. *If women do not receive information in their native language at the health station, they may rely on fake information from lay people who speak in their language (26 years old, employed)*.

*There are women who think that spiral is made of steel that can harm the body, and they are scared of it. They do not listen to you when you tell them that it is not a sharp thing*. *They need information from someone they can trust, particularly a health professional (37 years old in job training)*.

### Increasing multicultural communication skills of health providers

Some participants also suggested that health providers should be provided with multicultural communication training that would equip them with necessary skills to build good rapport and trust and understand women with different cultural backgrounds. This training would help in improving providers’ knowledge in provision of tailored information to immigrant women. In addition, employing people like midwives that have multi-cultural competence at health stations would help them gain trust of women.

*Women receive information from midwives who are mainly ethnic Norwegian*. *Some of these women have the perception that Norwegians are against having many children*. *They do not trust the information provided by the midwives because they think they have different culture regarding reproduction*. *Women need to receive information from someone they can trust*. *I think training health providers to increase their competence in multicultural health provision is important (26 years old, employed)*.

### Religious leaders as partners in contraception use

Participants explained the importance of engaging religious leaders in advocacy work for family planning. Many Somali women require go-ahead from religious leaders before they decide to seek contraception advice. The positive role of religious leaders in family planning is reported by participants to be critical in contraceptive use and they can be important facilitators to contraception use.

*Some women do not take their decisions regarding contraception until religious leaders approve that decision*. *It is important for religious leaders to be part of the awareness campaign (28 years old, employed)*.

## Community based facilitators

### Learning the language

Participants reported that knowledge about the local language is prerequisite for communication with health providers and it would make it easy for women to seek information about contraception and understand health advices. Some participants stressed the importance of health information about contraception particularly for newly arrived women. This is because participants believe that women are more vulnerable to unintended pregnancies in the first few years after arrival before they learn the language.

*Before I have learned the language, I was very scared and suspicious and I had no trust in the information I was given*. *When I learned the language, I started asking for information about contraception from the midwives. (46 years old in job training)*.

### Empowering women

Majority of the study participants emphasized the importance of empowering women and making them aware of risks related to pregnancy and short birth space intervals. According to participants, women should be empowered to be the ultimate decision-makers about their health and wellbeing including their reproduction rights.

*I think women, should be more conscious, to the problems that they may experience if they give birth closely spaced children*. *Men want many children because they do not go through the pain and difficulties associated with child bearing and care. (46 years old, unemployed)*

## Discussion

This study explored barriers and facilitators to contraception use among Somali immigrant women in Oslo, Norway. The study found that Somali women in Norway do not have their contraceptive needs met. System-related barriers, including the lack of appropriate information about the safety and accessibility of modern contraception, were among the barriers to contraceptive use. This finding may explain why immigrant and refugee women in Europe have higher rates of unintended pregnancy and abortion than non-immigrant women [5–9]. Many Somali women are skeptical about the modern contraception, which they associate with cancer, vitamin deficiency and infertility. This skepticism can be considered a direct consequence of a lack of appropriate information about modern contraception. Information provision requires a cultural understanding between immigrant women and health providers, with a culturally competent interpreter in place. Moreover, it may not be easy for many Somali women to discuss contraception and sexual health with a male doctor or through a male interpreter [32], which may limit their ability to discuss all their concerns with the male provider, and this compromises their right to take informed decisions. Training health providers for provision of appropriate and culturally tailored information about contraceptives is required to increase the contraception uptake of Somali immigrant women.

The study shows socio-cultural barriers mainly religious misperceptions that influence the decision of many Somali women in Norway to use contraception. This finding is in-line with prior finding among Muslim women in Africa whereby 66% of women’s decisions toward contraception is influenced by religion [33]. In Norway, this finding is unexpected given the fact that Somali immigrant women interact with many other Muslim communities that are knowledgeable about Islam and its position in family planning. Predominantly oral society, there are plenty of religious myths that thrive amongst them. For example, Somalis believe that female genital mutilation (FGM) is obligated by Islam, while many Muslim communities in Norway are not only totally unfamiliar with the practice, but also considered it un-Islamic. A study in USA found very low uptake of contraceptives among Somali women which was associated with religious beliefs [34]. However, in most Muslim countries, Imams support the use of modern contraception [35]. A study in Nigeria found that the exposure to family planning messages from religious leaders increased modern contraceptive use among Muslim women [33]. Campaigns against FGM in Norway have long used religious leaders as agent of change, which led to attitude change toward the practice among Somali immigrants [36]. Given the influential positions of religious leaders, the strategies to increase contraceptive uptake should engage religious leaders in advocacy work.

In this study, male partner’s resistance to family planning was another hurdle to contraception use. Participants of this study reported that there is a concern among women that if a woman fail to reproduce as many children as her husband wants, the husband is likely to abandon her to another woman who is willing to meet his fertility demand. A study in Tanzania reported that women using contraception against the wishes of the husbands could lead to violence or divorce of the woman [37], which support the Somali women’s concern. Our earlier quantitative study found that 20% of Somali women in Oslo have never discussed contraception with their husbands [31]. In a study on FGM amongst Somalis in Sweden asking men and women separately about potential perpetrators of FGM, men claimed that the practice is encouraged by women while women reported the contrary; after being brought together to discuss the issue and it became clear they had never discussed the issue. Similar situation may be true that Somali women and men do not discuss the contraception use; women may believe that men would not allow contraception use, while otherwise could be true with men. Nonetheless, Somalis are patriarchal society where men determines most of the family issues. A prior study of Somali women in Finland argued that if Somali men were educated about the importance of family planning, there might be a transformation of values and perceptions about birth control [24]. In sub-Saharan African, the majority of women considered modern contraception acceptable, but they require the permission of their partners before they actually adopted a modern method [38]. Consistent with this, a participant in our study suggested that there are many Somali women in Norway whose personal conviction is insufficient to ensure the uptake of modern contraceptive methods. Male involvement in family planning can increase uptake and continuation of contraceptive use by improving spousal communication [24, 38]. Therefore, any intervention to increase contraception use among Somali women in Oslo should educate women and men equally about family planning, and promote partner communication regarding contraceptive methods.

Participants have presented their views about facilitators to contraception use including participation of religious leaders in awareness creation campaigns. Many Somali women perceive contraception as against their religious teaching. This could be because some prominent Somali Imams speak against modern contraception. Contrastingly, in Arabic speaking Muslim countries such as Jordan, 82% of male religious leaders and 98% of female religious leaders believe that Islam encourages family planning [35]. Similarly, Muslim nations have actively engaged Islamic scholars to advocate for maternal and child health, including modern contraception [39, 40]. Study participants suggested that awareness campaigns aimed to change the perception of Somali immigrant women and men towards contraception should engage religious leaders in awareness programs. This engagement should go hand in hand with building capacity to enhance religious leaders’ knowledge on family planning methods.

Finally, participants think that empowering Somali women in decision making and autonomy for the decisions affecting their health and wellbeing is the most important factor affecting their contraceptive use. Women’s empowerment is defined as ‘the expansion of people’s ability to make strategic life choices in a context where this ability was previously denied to them’ [41] which is an important aspect affecting family planning and reproductive health outcomes of women. Empowering women through essential resources such as education and employment was associated with lower fertility, longer birth intervals and lower rates of unintended pregnancy [42]. While improving reproductive health rights of women is receiving growing attention in Norway [16], the barriers to contraception that Somali immigrant women experience are similar to barriers reported among women in different parts of Africa. Norwegian government efforts to promote reproductive health and rights of women should ensure that women’s individual resources such as education and employment are strengthened. Such efforts should enable women to make an informed contraceptive choice, which is consistent with their personal values, needs and beliefs.

This study had several limitations. The purposeful selection of study sample does not ensure that these populations were in fact representative of all Somali women in Norway. Moreover, study participants were women who have lived in Norway for a mean 17 years. Therefore, newly arrived women might have experienced different barriers that are not mentioned here. However, there are studies that investigated barriers to contraceptives among the Somali community, both in Somalia [22] and Somali immigrants in the West, which found similar results [43]. Despite these limitations, the result of this study contribute to the existing body of knowledge on utilization of contraception services among immigrant communities in Western countries. The findings demonstrate that, despite the availability and the equal access of contraceptive methods to all women in Norway, predominantly socio-cultural barriers impede Somali women’s contraceptive usage. The study findings underline that the availability of contraceptive methods in isolation is insufficient to ensure uptake amongst immigrant Somali women. Socio-cultural factors, such as the social pressure of having many children, men’s attitude toward contraception and religious misperceptions toward modern contraceptives, must be addressed.

## Conclusion

Family planning is affected by many factors operating at the level of the individual, family, peer group, community, institution, and policy environment. Eliminating the barriers preventing immigrant women from receiving modern contraception has important public health implications, including decreasing unplanned pregnancies and induced abortions. Participants had a good understanding of the importance of contraceptive methods for the health of the mother and the child. Although the majority had good knowledge of modern contraception and had experienced using it, they made it clear that Somali women face barriers to access. In order to successfully meet women's modern contraceptive needs, policy makers, health providers and civil society organizations should consider the barriers, and implement the facilitators reported by this study. Steps that we suggest include: the provision of appropriate health information on contraception to women, especially in early years of their arrival in the country; subsidy on contraceptive methods; encouraging partner communication in family planning; training health providers to improve their multicultural competence; and engaging religious leaders in advocacy work. Moreover, broader research is recommended that assesses the barriers at each level (individual, family, peer group, community, institutional, and the policy environment) to determine which interventions are best suited to increase Somali immigrant women’s contraception use. While health providers and policy makers should address system related barriers, civil society and community organizations should promote women’s empowerment and encourage partner’s discussion on family planning to counteract community based barriers. Further, given the influential positions of religious leaders, the strategies to increase contraceptive uptake should engage religious leaders in advocacy work. Finally, women’s low level of decision making power within families is a major barrier in contraception utilization affecting the health and development of immigrant women and thus, it should be addressed through multiple fronts.

## List of abbreviations

WHO: World Health Organization
UN: United Nations
