# Supplementary material for: Barriers and facilitators to contraceptive use among Somali immigrant women in Oslo: A qualitative study
Source: PLoS One. 2020 Mar 10;15(3):e0229916. doi: 10.1371/journal.pone.0229916 (PMC7064199; doi:10.1371/journal.pone.0229916)
Supplement: Attachment — Submitted filename: Recviewers Comments.docx [file pone.0229916.s001.docx]

**Title:** Barriers and facilitators to contraceptive use among Somali immigrant women in Oslo: A qualitative study

**Version Date:** 15^th^ September, 2019

**Reviewer:** Dr. Evelyn Sakeah

**Reviewer’s Report:**

This manuscript addresses barriers and facilitators to contraceptive use among Somali immigrant women. The topic is of great importance in global health.

**Abstract**

1. State the statistics of unintended pregnancy and abortion among immigrant

Women

2. In what format did the interviews take: IDIs or FGDs or both?

**Introduction**

1. Why should I take your results as new and useful? Have similar studies be conducted in the study country? Justify why it was necessary for the study to be conducted-show the gaps the authors filled in this research.

**Methods**

1. Describe the study site
2. State interviewing technique: IDIs or FGD or both?

The paper would be much clearer if the following comments are addressed

a. How many FGDs or IDIs were conducted?

b. How many participants took part in each FGD or IDI?

b. What was the average duration of the FGDs or IDIs?
3. Study design should be clearly stated from the beginning. What do the authors mean by this statement: “We employed a qualitative and quantitative design in this study, and the consistency of the findings from the two methods (interviews and quantitative data)”
4. Under Methods and materials, show study site; study design , sample and sampling and then the training of fieldworkers
5. Would suggest you include under "Methodology" section, the "theory that guided this study" and "methodology" of the study then adding the "methods of data collection".

**Training and data collection**

1. Indicate the training given to interviewers and how long the training was conducted
2. Please also make clear whether key terms were translated into local languages to harmonize data collection between interviewers. Also, were the interviews audio recorded, transcribed and translated into English for analysis?
3. Did interviewers review transcriptions for accuracy and completeness and corrected classification by questions to facilitate the work of the coding by theme? If these were done the authors should clearly state this in the methods section.

**Sample and Sampling technique**

1. Indicate the sample and show the category of people interviewed

**Analysis**

“We employed a qualitative and quantitative design in this study, and the consistency of the findings from the two methods (interviews and quantitative data) have served to ensure the trustworthiness and credibility of the study's results.”

1. This is purely a qualitative study, why then you employ a quantitative design as well.

**Results**

1. Please be consistent- you either use “participant “or “pt” in your quotations.
2. It would have been good for authors to add the ages of the participants in the quotations. That would help readers understand the perspectives of the younger participants as against the older ones.

**Discussion**

1. Please rewrite the opening paragraph of the discussion by stating clearly the summary of the research findings
2. Statements like the one below should be put in the conclusion/ recommendation section:

“Given the influential positions of religious leaders, the strategies to increase contraceptive uptake should engage religious leaders in advocacy work.”

**Quality of Written English:**

1. Suitable for publication with minor editing

**Ethical Approval**

1. Please add some more information on the processes related to the "ethical considerations", also add the IRB approval numbers to the text.

**Limitations**

1. Indicate the weaknesses of the study.
2. How did the authors prevent subjectivity in the conduct of this study?

**Data Availability**

1. Authors did not declare data availability
