# Supplementary material for: Barriers and facilitators to contraceptive use among Somali immigrant women in Oslo: A qualitative study
Source: PLoS One. 2020 Mar 10;15(3):e0229916. doi: 10.1371/journal.pone.0229916 (PMC7064199; doi:10.1371/journal.pone.0229916)
Supplement: Attachment — Submitted filename: Response to reviewers.docx [file pone.0229916.s002.docx]

**Title:** Barriers and facilitators to contraceptive use among Somali immigrant women in Oslo: A qualitative study

**Version Date:** 15^th^ September, 2019

**Reviewer:** Dr. Evelyn Sakeah

**Reviewer’s Report:**

This manuscript addresses barriers and facilitators to contraceptive use among Somali immigrant women. The topic is of great importance in global health.

**Abstract**

1. State the statistics of unintended pregnancy and abortion among immigrant

Women

Thanks to the reviewer for important comments. Done. Look the abstract, background section, from line 3 to 6

2. In what format did the interviews take: IDIs or FGDs or both?

The study used unstructured in-depth interview. Done, look the abstract, methods section, first line

3. **Introduction**
1. Why should I take your results as new and useful? Have similar studies be conducted in the study country? Justify why it was necessary for the study to be conducted-show the gaps the authors filled in this research. Such study has never been done in Norway

Done, the final paragraph of the Introduction section

**Methods**

1. Describe the study site

Done. Look the methods section, first paragraph, line 1-3

2. State interviewing technique: IDIs or FGD or both?

The study used unstructured interview. Look the methods section, from 3-6

The paper would be much clearer if the following comments are addressed

a. How many FGDs or IDIs were conducted?

The study used un-structured interviews involving 21 interviews. Look the methods section, from line 6-7

b. How many participants took part in each FGD or IDI?

The study used un-structured in-depth interviews involving 21 interviews. Look the methods section, from line 6-7. There was no FGDs.

b. What was the average duration of the FGDs or IDIs?

The interviews lasted from 45-60 minutes. Done, look methods section, 2^nd^ paragraph, line 17-18

3. Study design should be clearly stated from the beginning.

Done, look the methods section, first paragraph

What do the authors mean by this statement: “We employed a qualitative and quantitative design in this study, and the consistency of the findings from the two methods (interviews and quantitative data)”

This study is a part of a broad project that used quantitative and qualitative design. The statement is now rephrased.

4. Under Methods and materials, show study site; study design , sample and sampling and then the training of fieldworkers

Done. Data was collected by experienced researchers, therefore, fieldworkers were not used; thus, no training was provided.

5. Would suggest you include under "Methodology" section, the "methodology" of the study then adding the "methods of data collection".

Done. Look the methodology section. We organized it as ‘ **Sample and Sampling technique, Data collection, and Analysis’** as suggested by the reviewer.

**Training and data collection**

1. Indicate the training given to interviewers and how long the training was conducted

Data were collected by experienced researchers; first and second author, both are native Somali speakers; Somali language was also the native language of all study participants.

2. Please also make clear whether key terms were translated into local languages to harmonize data collection between interviewers. Also, were the interviews audio recorded,

The interviews were conducted in Somali, which was the native language of both researchers and participants. Yes, interviews were audio recorded. Look methods section, line 18-19

3. Transcribed and translated into English for analysis?

Yes. Look the analysis, line 1-2

4. Did interviewers review transcriptions for accuracy and completeness and corrected classification by questions to facilitate the work of the coding by theme?

Yes, that was the case. Look the analysis section, line 2-3

**Sample and Sampling technique**

1. Indicate the sample and show the category of people interviewed

Done. Look at the Methods section, first paragraph, line 5-6

**Analysis**

“We employed a qualitative and quantitative design in this study, and the consistency of the findings from the two methods (interviews and quantitative data) have served to ensure the trustworthiness and credibility of the study's results.”

1. This is purely a qualitative study, why then you employ a quantitative design as well.

Thanks for taking this into our attention. Yes, this study is a qualitative, but we did a quantitative part as well, which was published earlier in this journal. We clarified the statement more clearly in the paper. Look analysis section, line 9-10

**Results**

1. Please be consistent- you either use “participant “or “pt” in your quotations.

Corrected. We changed them to ‘participants’

2. It would have been good for authors to add the ages of the participants in the quotations. That would help readers understand the perspectives of the younger participants as against the older ones.

As participants were only 21 persons, we couldn’t add the age in the quotations, for fear that, with age in the quotation, some participants may become identifiable. The age of the participants are shown in table two.

**Discussion**

1. Please rewrite the opening paragraph of the discussion by stating clearly the summary of the research findings

Done. Look the discussion section, line 1-5

2. Statements like the one below should be put in the conclusion/ recommendation section:

“Given the influential positions of religious leaders, the strategies to increase contraceptive uptake should engage religious leaders in advocacy work.”

Thanks for the very important suggestion. Done, look the conclusion section. Line 18-19

**Quality of Written English:**

1. Suitable for publication with minor editing

Thanks, the language was crosschecked and edited where necessary.

**Ethical Approval**

1. Please add some more information on the processes related to the "ethical considerations", also add the IRB approval numbers to the text.

Done. Look the ethical approval section

**Limitations**

1. Indicate the weaknesses of the study.

Look the discussion section, final paragraph

2. How did the authors prevent subjectivity in the conduct of this study?

To avoid biases, we have employed two important steps:

1- We used three researchers to code the data.
2- We allowed three members from the participants to review the results and verify that what they said was stated correctly.

This was clearly mentioned in the last two lines of the ‘analysis section’

**Data Availability**

1. Authors did not declare data availability

There are legal restrictions on sharing the de-identified data set, as sharing data require approval by Norwegian Regional Committee for medical and health research ethics (REK). Data is available on request from Norwegian Institute of Public Health, but the provision of the data should be approved by REK. They can be contacted via: [post@helseforskning.etikkom.no](mailto:post@helseforskning.etikkom.no)
