# Supplementary material for: Barriers and facilitators to contraceptive use among Somali immigrant women in Oslo: A qualitative study
Source: PLoS One. 2020 Mar 10;15(3):e0229916. doi: 10.1371/journal.pone.0229916 (PMC7064199; doi:10.1371/journal.pone.0229916)
Supplement: Attachment — Submitted filename: Response to editor.docx [file pone.0229916.s003.docx]

Response to editor’s comments

1. Line 6, page 2 - expression should be "these findings" not "these finding"

Done line 7, page 2
2. Line 12, page 4 - expression should be "in unintended pregnancies"

Done line 12, page 4
3. Last but one line on page 4 - expression "and they" should have been "that". Also, it is not clear if it is "the visit" that is free or "the contraceptives"? it does not come out clearly...

Done, page 4, line 23
4. Line 1, page 6 - This "is" mirrors - "is" should be deleted...

Done, page 6, line 1
5. Line 7, page 8 - until researchers "become" - "become" should be in the past tense

Done, page 8, line 7.
6. Last but one and last lines of page 9 - "the findings from a quantitative part of this study" - it would be great to clarify which findings these are...

Done, page 9, line 21 to page 10, line 2.
7. Lines 2 - 6 under results on page 10 - could you elaborate a bit on the basis upon which these conclusions were made

The statement was removed as we found it not fitting well there.
8. Pages 10 and 11- It will be good you provide an overview of what is meant by "system related or system based barriers or facilitators" and "socio-cultural or community based facilitators or barriers".

Done. Short clarification of the terms (socio-cultural and system based) was added. Look page 11, line 4-5, and page 14, line 16-18

Also I am not sure why "perceived side-effects" and "religious leaders as partners" would be "system related". it will be great you clarify how you arrived at those assumptions.

Perceived side effect and the resultant fear of contraceptives are basically due to lack of appropriate information about contraceptives. Provision of relevant information to immigrants is the responsibility of the health system providers. Similarly, we placed ‘religious leaders as partners’ within the system related facilitators. In Norway, religious leaders are included in health awareness campaigns such as elimination of Female Genital Mutilation. Also, different municipalities provide fund to civil society organizations to prevent forced marriage and other social norms, and municipalities often encourage inclusion of religious leaders in the awareness efforts. For that, they are part of the preventive health system and in this paper, we think that their advocacy regarding promotion of contraception use will help immigrant women accept modern contraceptive methods

9. Describing participants whose quotes were used. It is necessary to provide some information on your participants. A reader will not be running up and down your manuscript to be referring to such details elsewhere...You could present "Participant 20"as "46 year old mother in job"training" and "Participant 19" as "44 year old mother in job-training" .... please do it similarly for other quotes...

Done. Throughout the paper
10. For each of the "sub-themes", provide "self explanatory statements". E.g. "Cost" on page 14 could be something like "High cost of contraceptives"... "religion" e.g. "religious beliefs not favoring contraceptive use"... do that for the other sub-themes and themes. Also the finding "high cost of contraception was reported..." on page 14 seems to conflict with information you provided in the background - so please address this disparity in your discussions.

Done. It was mainly based on misinformation of the participant, and we removed it to eliminate misunderstandings.
11. Page 14 - the statement "One participant, who was knowledgeable about Islam has reasoned why women should plan their pregnancies" is not clear, so some revision will help

Done. page 15, line 11-12
12. Page 17 - define or describe what you refer to as "multicultural communication"

Done. Page 18, line 2-5
13. Page 24 - the statement "there are number of studies in this community either in Africa [22] or among immigrants in the West" is not clear, so revise

Done. Page 24, line 11-15
14. Revise the following in the REFERENCE SECTION: numbers 10, 28, 35, 37, 38, and check if there are others that do not conform to the requirements of the journal

Done. We checked all references and corrected where necessary.
15. There are still a few grammatical errors here and there.... please do a "thorough sweep-through" to ensure you clear them all...

Done. Proof-reading by native speaker was done.
