# Supplementary material for: Barriers and facilitators to contraceptive use among Somali immigrant women in Oslo: A qualitative study
Source: PLoS One. 2020 Mar 10;15(3):e0229916. doi: 10.1371/journal.pone.0229916 (PMC7064199; doi:10.1371/journal.pone.0229916)
Supplement: Peer review history

# Peer review: Decision Letter 0

**DOI:** 10.1371/journal.pone.0229916.r001

**Authors:** Yeetey Akpe Kwesi Enuameh

29 Oct 2019

PONE-D-19-23365

Barriers and facilitators to contraceptive use among Somali immigrant women in Oslo: A qualitative study

PLOS ONE

Dear Dr Gele,

Thank you for submitting your manuscript to PLOS ONE. After careful consideration, we feel that it has merit but does not fully meet PLOS ONE’s publication criteria as it currently stands. Therefore, we invite you to submit a revised version of the manuscript that addresses the points raised during the review process.

We would appreciate receiving your revised manuscript by Dec 13 2019 11:59PM. When you are ready to submit your revision, log on to https://www.editorialmanager.com/pone/ and select the 'Submissions Needing Revision' folder to locate your manuscript file.

If you would like to make changes to your financial disclosure, please include your updated statement in your cover letter.

To enhance the reproducibility of your results, we recommend that if applicable you deposit your laboratory protocols in protocols.io, where a protocol can be assigned its own identifier (DOI) such that it can be cited independently in the future. For instructions see: http://journals.plos.org/plosone/s/submission-guidelines#loc-laboratory-protocols

Please include the following items when submitting your revised manuscript:

- A rebuttal letter that responds to each point raised by the academic editor and reviewer(s). This letter should be uploaded as separate file and labeled 'Response to Reviewers'.
- A marked-up copy of your manuscript that highlights changes made to the original version. This file should be uploaded as separate file and labeled 'Revised Manuscript with Track Changes'.
- An unmarked version of your revised paper without tracked changes. This file should be uploaded as separate file and labeled 'Manuscript'.

Please note while forming your response, if your article is accepted, you may have the opportunity to make the peer review history publicly available. The record will include editor decision letters (with reviews) and your responses to reviewer comments. If eligible, we will contact you to opt in or out.

We look forward to receiving your revised manuscript.

Kind regards,

Yeetey Akpe Kwesi Enuameh, MD, MSc, DrPH

Academic Editor

PLOS ONE

Journal Requirements:

1.

When submitting your revision, we need you to address these additional requirements.

Please ensure that your manuscript meets PLOS ONE's style requirements, including those for file naming. The PLOS ONE style templates can be found at

http://www.journals.plos.org/plosone/s/file?id=wjVg/PLOSOne_formatting_sample_main_body.pdf and http://www.journals.plos.org/plosone/s/file?id=ba62/PLOSOne_formatting_sample_title_authors_affiliations.pdf

2. Please amend your current ethics statement to address the following concerns: Please explain why was written consent was not obtained, how you recorded/documented participant consent, and if the ethics committees/IRBs approved this consent procedure.

3. Note from Associate Editor Nancy Beam (nbeam@plos.org): PLOS ONE considers qualitative and mixed-methods studies for publication. We recommend that authors use the COREQ checklist, or other relevant checklists listed by the Equator Network, such as the SRQR, to ensure complete reporting (http://journals.plos.org/plosone/s/submission-guidelines#loc-qualitative-research).

In general, we would expect qualitative studies to include the following: 1) defined objectives or research questions; 2) description of the sampling strategy, including rationale for the recruitment method, participant inclusion/exclusion criteria and the number of participants recruited; 3) detailed reporting of the data collection procedures; 4) data analysis procedures described in sufficient detail to enable replication; 5) a discussion of potential sources of bias; and 6) a discussion of limitations.  In your role as Academic Editor, we appreciate your consideration of whether the manuscript meets reporting standards in the field, in addition to the journal’s other publication criteria (https://journals.plos.org/plosone/s/criteria-for-publication). Please feel free to email me to discuss the work further.

4. 

We note that you have indicated that data from this study are available upon request. PLOS only allows data to be available upon request if there are legal or ethical restrictions on sharing data publicly. For information on unacceptable data access restrictions, please see http://journals.plos.org/plosone/s/data-availability#loc-unacceptable-data-access-restrictions.

In your revised cover letter, please address the following prompts:

a) If there are ethical or legal restrictions on sharing a de-identified data set, please explain them in detail (e.g., data contain potentially identifying or sensitive patient information) and who has imposed them (e.g., an ethics committee). Please also provide contact information for a data access committee, ethics committee, or other institutional body to which data requests may be sent.

b) If there are no restrictions, please upload the minimal anonymized data set necessary to replicate your study findings as either Supporting Information files or to a stable, public repository and provide us with the relevant URLs, DOIs, or accession numbers. Please see http://www.bmj.com/content/340/bmj.c181.long for guidelines on how to de-identify and prepare clinical data for publication. For a list of acceptable repositories, please see http://journals.plos.org/plosone/s/data-availability#loc-recommended-repositories.

We will update your Data Availability statement on your behalf to reflect the information you provide.

5. Please include your tables as part of your main manuscript and remove the individual files. Please note that supplementary tables (should remain/ be uploaded) as separate "supporting information" files

Additional Editor Comments (if provided):

Please address comments provided by the reviewer.

Also, though socio-demographics of respondents were added to the appendices, please add some relevant information to the participants' details in the narrative section instead of just participant 1, etc.

[Note: HTML markup is below. Please do not edit.]

Reviewers' comments:

Reviewer's Responses to Questions

**Comments to the Author**

1. Is the manuscript technically sound, and do the data support the conclusions?

The manuscript must describe a technically sound piece of scientific research with data that supports the conclusions. Experiments must have been conducted rigorously, with appropriate controls, replication, and sample sizes. The conclusions must be drawn appropriately based on the data presented. 

Reviewer #1: Yes

**********

2. Has the statistical analysis been performed appropriately and rigorously? 

Reviewer #1: N/A

**********

3. Have the authors made all data underlying the findings in their manuscript fully available?

The PLOS Data policy requires authors to make all data underlying the findings described in their manuscript fully available without restriction, with rare exception (please refer to the Data Availability Statement in the manuscript PDF file). The data should be provided as part of the manuscript or its supporting information, or deposited to a public repository. For example, in addition to summary statistics, the data points behind means, medians and variance measures should be available. If there are restrictions on publicly sharing data—e.g. participant privacy or use of data from a third party—those must be specified.

Reviewer #1: No

**********

4. Is the manuscript presented in an intelligible fashion and written in standard English?

PLOS ONE does not copyedit accepted manuscripts, so the language in submitted articles must be clear, correct, and unambiguous. Any typographical or grammatical errors should be corrected at revision, so please note any specific errors here.

Reviewer #1: Yes

**********

5. Review Comments to the Author

Please use the space provided to explain your answers to the questions above. You may also include additional comments for the author, including concerns about dual publication, research ethics, or publication ethics. (Please upload your review as an attachment if it exceeds 20,000 characters)

Reviewer #1: Title: Barriers and facilitators to contraceptive use among Somali immigrant women in Oslo: A qualitative study

Version Date: 15th September, 2019

Reviewer: Dr. Evelyn Sakeah

Reviewer’s Report:

This manuscript addresses barriers and facilitators to contraceptive use among Somali immigrant women. The topic is of great importance in global health.

Abstract

1. State the statistics of unintended pregnancy and abortion among immigrant

Women

2. In what format did the interviews take: IDIs or FGDs or both?

Introduction

1. Why should I take your results as new and useful? Have similar studies be conducted in the study country? Justify why it was necessary for the study to be conducted-show the gaps the authors filled in this research.

Methods

1. Describe the study site

2. State interviewing technique: IDIs or FGD or both?

The paper would be much clearer if the following comments are addressed

a. How many FGDs or IDIs were conducted?

b. How many participants took part in each FGD or IDI?

b. What was the average duration of the FGDs or IDIs?

3. Study design should be clearly stated from the beginning. What do the authors mean by this statement: “We employed a qualitative and quantitative design in this study, and the consistency of the findings from the two methods (interviews and quantitative data)”

4. Under Methods and materials, show study site; study design , sample and sampling and then the training of fieldworkers

5. Would suggest you include under "Methodology" section, the "theory that guided this study" and "methodology" of the study then adding the "methods of data collection".

Training and data collection

1. Indicate the training given to interviewers and how long the training was conducted

2. Please also make clear whether key terms were translated into local languages to harmonize data collection between interviewers. Also, were the interviews audio recorded, transcribed and translated into English for analysis?

3. Did interviewers review transcriptions for accuracy and completeness and corrected classification by questions to facilitate the work of the coding by theme? If these were done the authors should clearly state this in the methods section.

Sample and Sampling technique

1. Indicate the sample and show the category of people interviewed

Analysis

“We employed a qualitative and quantitative design in this study, and the consistency of the findings from the two methods (interviews and quantitative data) have served to ensure the trustworthiness and credibility of the study's results.”

1. This is purely a qualitative study, why then you employ a quantitative design as well.

Results

1. Please be consistent- you either use “participant “or “pt” in your quotations.

2. It would have been good for authors to add the ages of the participants in the quotations. That would help readers understand the perspectives of the younger participants as against the older ones.

Discussion

1. Please rewrite the opening paragraph of the discussion by stating clearly the summary of the research findings

2. Statements like this should be put in the recommendation section: “Given the influential positions of religious leaders, the strategies to increase contraceptive uptake should engage religious leaders in advocacy work.”

Quality of Written English:

1. Suitable for publication with minor editing

Ethical Approval

1. Please add some more information on the processes related to the "ethical considerations", also add the IRB approval numbers to the text.

Limitations

1. Indicate the weaknesses of the study.

2. How did the authors prevent subjectivity in the conduct of this study?

Data Availability

1. Authors did not declare data availability

**********

6. PLOS authors have the option to publish the peer review history of their article (what does this mean?). If published, this will include your full peer review and any attached files.

If you choose “no”, your identity will remain anonymous but your review may still be made public.

**Do you want your identity to be public for this peer review?** For information about this choice, including consent withdrawal, please see our Privacy Policy.

Reviewer #1: None

[NOTE: If reviewer comments were submitted as an attachment file, they will be attached to this email and accessible via the submission site. Please log into your account, locate the manuscript record, and check for the action link "View Attachments". If this link does not appear, there are no attachment files to be viewed.]

While revising your submission, please upload your figure files to the Preflight Analysis and Conversion Engine (PACE) digital diagnostic tool, https://pacev2.apexcovantage.com/. PACE helps ensure that figures meet PLOS requirements. To use PACE, you must first register as a user. Registration is free. Then, login and navigate to the UPLOAD tab, where you will find detailed instructions on how to use the tool. If you encounter any issues or have any questions when using PACE, please email us at figures@plos.org. Please note that Supporting Information files do not need this step.

Attachment Submitted filename: Recviewers Comments.docx Click here for additional data file.

# Author comment: Author response to Decision Letter 0

**DOI:** 10.1371/journal.pone.0229916.r002

11 Nov 2019

Title: Barriers and facilitators to contraceptive use among Somali immigrant women in Oslo: A qualitative study

Version Date: 15th September, 2019

Reviewer: Dr. Evelyn Sakeah

Reviewer’s Report:

This manuscript addresses barriers and facilitators to contraceptive use among Somali immigrant women. The topic is of great importance in global health.

Abstract

1. State the statistics of unintended pregnancy and abortion among immigrant

Women 

Thanks to the reviewer for important comments. Done. Look the abstract, background section, from line 3 to 6

2. In what format did the interviews take: IDIs or FGDs or both? 

The study used unstructured in-depth interview. Done, look the abstract, methods section, first line

3. Introduction

1. Why should I take your results as new and useful? Have similar studies be conducted in the study country? Justify why it was necessary for the study to be conducted-show the gaps the authors filled in this research. Such study has never been done in Norway

Done, the final paragraph of the Introduction section

Methods

1. Describe the study site 

Done. Look the methods section, first paragraph, line 1-3

2. State interviewing technique: IDIs or FGD or both? 

The study used unstructured interview. Look the methods section, from 3-6

The paper would be much clearer if the following comments are addressed

a. How many FGDs or IDIs were conducted?

The study used un-structured interviews involving 21 interviews. Look the methods section, from line 6-7

b. How many participants took part in each FGD or IDI?

The study used un-structured in-depth interviews involving 21 interviews. Look the methods section, from line 6-7. There was no FGDs.

b. What was the average duration of the FGDs or IDIs? 

The interviews lasted from 45-60 minutes. Done, look methods section, 2nd paragraph, line 17-18

3. Study design should be clearly stated from the beginning. 

Done, look the methods section, first paragraph

What do the authors mean by this statement: “We employed a qualitative and quantitative design in this study, and the consistency of the findings from the two methods (interviews and quantitative data)” 

This study is a part of a broad project that used quantitative and qualitative design. The statement is now rephrased. 

4. Under Methods and materials, show study site; study design , sample and sampling and then the training of fieldworkers

Done. Data was collected by experienced researchers, therefore, fieldworkers were not used; thus, no training was provided. 

5. Would suggest you include under "Methodology" section, the "methodology" of the study then adding the "methods of data collection".

Done. Look the methodology section. We organized it as ‘ Sample and Sampling technique, Data collection, and Analysis’ as suggested by the reviewer.

Training and data collection 

1. Indicate the training given to interviewers and how long the training was conducted

 Data were collected by experienced researchers; first and second author, both are native Somali speakers; Somali language was also the native language of all study participants. 

2. Please also make clear whether key terms were translated into local languages to harmonize data collection between interviewers. Also, were the interviews audio recorded, 

The interviews were conducted in Somali, which was the native language of both researchers and participants. Yes, interviews were audio recorded. Look methods section, line 18-19

3. Transcribed and translated into English for analysis?

Yes. Look the analysis, line 1-2

4. Did interviewers review transcriptions for accuracy and completeness and corrected classification by questions to facilitate the work of the coding by theme? 

Yes, that was the case. Look the analysis section, line 2-3

Sample and Sampling technique

1. Indicate the sample and show the category of people interviewed

Done. Look at the Methods section, first paragraph, line 5-6

Analysis

“We employed a qualitative and quantitative design in this study, and the consistency of the findings from the two methods (interviews and quantitative data) have served to ensure the trustworthiness and credibility of the study's results.”

1. This is purely a qualitative study, why then you employ a quantitative design as well.

Thanks for taking this into our attention. Yes, this study is a qualitative, but we did a quantitative part as well, which was published earlier in this journal. We clarified the statement more clearly in the paper. Look analysis section, line 9-10

Results

1. Please be consistent- you either use “participant “or “pt” in your quotations. 

Corrected. We changed them to ‘participants’

2. It would have been good for authors to add the ages of the participants in the quotations. That would help readers understand the perspectives of the younger participants as against the older ones.

As participants were only 21 persons, we couldn’t add the age in the quotations, for fear that, with age in the quotation, some participants may become identifiable. The age of the participants are shown in table two.

Discussion

1. Please rewrite the opening paragraph of the discussion by stating clearly the summary of the research findings

Done. Look the discussion section, line 1-5

2. Statements like the one below should be put in the conclusion/ recommendation section: 

“Given the influential positions of religious leaders, the strategies to increase contraceptive uptake should engage religious leaders in advocacy work.”

Thanks for the very important suggestion. Done, look the conclusion section. Line 18-19

Quality of Written English: 

1. Suitable for publication with minor editing

Thanks, the language was crosschecked and edited where necessary.

Ethical Approval

1. Please add some more information on the processes related to the "ethical considerations", also add the IRB approval numbers to the text.

Done. Look the ethical approval section

Limitations

1. Indicate the weaknesses of the study.

Look the discussion section, final paragraph

2. How did the authors prevent subjectivity in the conduct of this study?

To avoid biases, we have employed two important steps:

1- We used three researchers to code the data. 

2- We allowed three members from the participants to review the results and verify that what they said was stated correctly.

This was clearly mentioned in the last two lines of the ‘analysis section’

Data Availability

1. Authors did not declare data availability

There are legal restrictions on sharing the de-identified data set, as sharing data require approval by Norwegian Regional Committee for medical and health research ethics (REK). Data is available on request from Norwegian Institute of Public Health, but the provision of the data should be approved by REK. They can be contacted via: post@helseforskning.etikkom.no

Attachment Submitted filename: Response to reviewers.docx Click here for additional data file.

# Peer review: Decision Letter 1

**DOI:** 10.1371/journal.pone.0229916.r003

**Authors:** Yeetey Akpe Kwesi Enuameh

8 Jan 2020

PONE-D-19-23365R1

Barriers and facilitators to contraceptive use among Somali immigrant women in Oslo: A qualitative study

PLOS ONE

Dear Abdi Gele,

Thank you for submitting your manuscript to PLOS ONE. After careful consideration, we feel that it has merit but does not fully meet PLOS ONE’s publication criteria as it currently stands. Therefore, we invite you to submit a revised version of the manuscript that addresses the points raised during the review process.

We would appreciate receiving your revised manuscript by Feb 22 2020 11:59PM. When you are ready to submit your revision, log on to https://www.editorialmanager.com/pone/ and select the 'Submissions Needing Revision' folder to locate your manuscript file.

If you would like to make changes to your financial disclosure, please include your updated statement in your cover letter.

To enhance the reproducibility of your results, we recommend that if applicable you deposit your laboratory protocols in protocols.io, where a protocol can be assigned its own identifier (DOI) such that it can be cited independently in the future. For instructions see: http://journals.plos.org/plosone/s/submission-guidelines#loc-laboratory-protocols

Please include the following items when submitting your revised manuscript:

- A rebuttal letter that responds to each point raised by the academic editor and reviewer(s). This letter should be uploaded as separate file and labeled 'Response to Reviewers'.
- A marked-up copy of your manuscript that highlights changes made to the original version. This file should be uploaded as separate file and labeled 'Revised Manuscript with Track Changes'.
- An unmarked version of your revised paper without tracked changes. This file should be uploaded as separate file and labeled 'Manuscript'.

Please note while forming your response, if your article is accepted, you may have the opportunity to make the peer review history publicly available. The record will include editor decision letters (with reviews) and your responses to reviewer comments. If eligible, we will contact you to opt in or out.

We look forward to receiving your revised manuscript.

Kind regards,

Yeetey Akpe Kwesi Enuameh, MD, MSc, DrPH

Academic Editor

PLOS ONE

Additional Editor Comments (if provided):

Nice effort addressing issues raised by reviewer... there are a few more things to add that emerged post the first review. NB: In your subsequent publications, please number each of the lines to make it easier for reviewers to make references to portions of your work...

1. Line 6, page 2 - expression should be "these findings" not "these finding"

2. Line 12, page 4 - expression should be "in unintended pregnancies"

3. Last but one line on page 4 - expression "and they" should have been "that". Also, it is not clear if it is "the visit" that is free or "the contraceptives"? it does not come out clearly...

4. Line 1, page 6 - This "is" mirrors - "is" should be deleted...

5. Line 7, page 8 - until researchers "become" - "become" should be in the past tense

6. Last but one and last lines of page 9 - "the findings from a quantitative part of this study" - it would be great to clarify which findings these are...

7. Lines 2 - 6 under results on page 10 - could you elaborate a bit on the basis upon which these conclusions were made

8. Pages 10 and 11- It will be good you provide an overview of what is meant by "system related or system based barriers or facilitators" and "socio-cultural or community based facilitators or barriers". Also I am not sure why "perceived side-effects" and "religious leaders as partners" would be "system related". it will be great you clarify how you arrived at those assumptions.

9. Describing participants whose quotes were used. It is necessary to provide some information on your participants. A reader will not be running up and down your manuscript to be referring to such details elsewhere...You could present "Participant 20"as "46 year old mother in job"training" and "Participant 19" as "44 year old mother in job-training" .... please do it similarly for other quotes...

10. For each of the "sub-themes", provide "self explanatory statements". E.g. "Cost" on page 14 could be something like "High cost of contraceptives"... "religion" e.g. "religious beliefs not favoring contraceptive use"... do that for the other sub-themes and themes. Also the finding "high cost of contraception was reported..." on page 14 seems to conflict with information you provided in the background - so please address this disparity in your discussions.

11. Page 14 - the statement "One participant, who was knowledgeable about Islam has reasoned why women should plan their pregnancies" is not clear, so some revision will help

12. Page 17 - define or describe what you refer to as "multicultural communication"

13. Page 24 - the statement "there are number of studies in this community either in Africa [22] or among immigrants in the West" is not clear, so revise

14. Revise the following in the REFERENCE SECTION: numbers 10, 28, 35, 37, 38, and check if there are others that do not conform to the requirements of the journal

15. There are still a few grammatical errors here and there.... please do a "thorough sweep-through" to ensure you clear them all...

[Note: HTML markup is below. Please do not edit.]

Reviewers' comments:

Reviewer's Responses to Questions

**Comments to the Author**

1. If the authors have adequately addressed your comments raised in a previous round of review and you feel that this manuscript is now acceptable for publication, you may indicate that here to bypass the “Comments to the Author” section, enter your conflict of interest statement in the “Confidential to Editor” section, and submit your "Accept" recommendation.

Reviewer #1: All comments have been addressed

**********

2. Is the manuscript technically sound, and do the data support the conclusions?

The manuscript must describe a technically sound piece of scientific research with data that supports the conclusions. Experiments must have been conducted rigorously, with appropriate controls, replication, and sample sizes. The conclusions must be drawn appropriately based on the data presented. 

Reviewer #1: Yes

**********

3. Has the statistical analysis been performed appropriately and rigorously? 

Reviewer #1: N/A

**********

4. Have the authors made all data underlying the findings in their manuscript fully available?

The PLOS Data policy requires authors to make all data underlying the findings described in their manuscript fully available without restriction, with rare exception (please refer to the Data Availability Statement in the manuscript PDF file). The data should be provided as part of the manuscript or its supporting information, or deposited to a public repository. For example, in addition to summary statistics, the data points behind means, medians and variance measures should be available. If there are restrictions on publicly sharing data—e.g. participant privacy or use of data from a third party—those must be specified.

Reviewer #1: No

**********

5. Is the manuscript presented in an intelligible fashion and written in standard English?

PLOS ONE does not copyedit accepted manuscripts, so the language in submitted articles must be clear, correct, and unambiguous. Any typographical or grammatical errors should be corrected at revision, so please note any specific errors here.

Reviewer #1: Yes

**********

6. Review Comments to the Author

Please use the space provided to explain your answers to the questions above. You may also include additional comments for the author, including concerns about dual publication, research ethics, or publication ethics. (Please upload your review as an attachment if it exceeds 20,000 characters)

Reviewer #1: The article is well written and all comments have been addressed. The only concern is their unpreparedness to make the data available. However, they provided an explanation to that.

**********

7. PLOS authors have the option to publish the peer review history of their article (what does this mean?). If published, this will include your full peer review and any attached files.

If you choose “no”, your identity will remain anonymous but your review may still be made public.

**Do you want your identity to be public for this peer review?** For information about this choice, including consent withdrawal, please see our Privacy Policy.

Reviewer #1: Yes: Evelyn Sakeah

[NOTE: If reviewer comments were submitted as an attachment file, they will be attached to this email and accessible via the submission site. Please log into your account, locate the manuscript record, and check for the action link "View Attachments". If this link does not appear, there are no attachment files to be viewed.]

While revising your submission, please upload your figure files to the Preflight Analysis and Conversion Engine (PACE) digital diagnostic tool, https://pacev2.apexcovantage.com/. PACE helps ensure that figures meet PLOS requirements. To use PACE, you must first register as a user. Registration is free. Then, login and navigate to the UPLOAD tab, where you will find detailed instructions on how to use the tool. If you encounter any issues or have any questions when using PACE, please email us at figures@plos.org. Please note that Supporting Information files do not need this step.

# Author comment: Author response to Decision Letter 1

**DOI:** 10.1371/journal.pone.0229916.r004

21 Jan 2020

Response to editor’s comments

1. Line 6, page 2 - expression should be "these findings" not "these finding"

Done line 7, page 2

2. Line 12, page 4 - expression should be "in unintended pregnancies"

Done line 12, page 4

3. Last but one line on page 4 - expression "and they" should have been "that". Also, it is not clear if it is "the visit" that is free or "the contraceptives"? it does not come out clearly...

Done, page 4, line 23

4. Line 1, page 6 - This "is" mirrors - "is" should be deleted...

Done, page 6, line 1

5. Line 7, page 8 - until researchers "become" - "become" should be in the past tense

Done, page 8, line 7.

6. Last but one and last lines of page 9 - "the findings from a quantitative part of this study" - it would be great to clarify which findings these are...

Done, page 9, line 21 to page 10, line 2.

7. Lines 2 - 6 under results on page 10 - could you elaborate a bit on the basis upon which these conclusions were made

The statement was removed as we found it not fitting well there. 

8. Pages 10 and 11- It will be good you provide an overview of what is meant by "system related or system based barriers or facilitators" and "socio-cultural or community based facilitators or barriers". 

Done. Short clarification of the terms (socio-cultural and system based) was added. Look page 11, line 4-5, and page 14, line 16-18

Also I am not sure why "perceived side-effects" and "religious leaders as partners" would be "system related". it will be great you clarify how you arrived at those assumptions.

Perceived side effect and the resultant fear of contraceptives are basically due to lack of appropriate information about contraceptives. Provision of relevant information to immigrants is the responsibility of the health system providers. Similarly, we placed ‘religious leaders as partners’ within the system related facilitators. In Norway, religious leaders are included in health awareness campaigns such as elimination of Female Genital Mutilation. Also, different municipalities provide fund to civil society organizations to prevent forced marriage and other social norms, and municipalities often encourage inclusion of religious leaders in the awareness efforts. For that, they are part of the preventive health system and in this paper, we think that their advocacy regarding promotion of contraception use will help immigrant women accept modern contraceptive methods

9. Describing participants whose quotes were used. It is necessary to provide some information on your participants. A reader will not be running up and down your manuscript to be referring to such details elsewhere...You could present "Participant 20"as "46 year old mother in job"training" and "Participant 19" as "44 year old mother in job-training" .... please do it similarly for other quotes...

Done. Throughout the paper

10. For each of the "sub-themes", provide "self explanatory statements". E.g. "Cost" on page 14 could be something like "High cost of contraceptives"... "religion" e.g. "religious beliefs not favoring contraceptive use"... do that for the other sub-themes and themes. Also the finding "high cost of contraception was reported..." on page 14 seems to conflict with information you provided in the background - so please address this disparity in your discussions.

Done. It was mainly based on misinformation of the participant, and we removed it to eliminate misunderstandings.

11. Page 14 - the statement "One participant, who was knowledgeable about Islam has reasoned why women should plan their pregnancies" is not clear, so some revision will help

Done. page 15, line 11-12

12. Page 17 - define or describe what you refer to as "multicultural communication"

Done. Page 18, line 2-5

13. Page 24 - the statement "there are number of studies in this community either in Africa [22] or among immigrants in the West" is not clear, so revise

Done. Page 24, line 11-15

14. Revise the following in the REFERENCE SECTION: numbers 10, 28, 35, 37, 38, and check if there are others that do not conform to the requirements of the journal

Done. We checked all references and corrected where necessary. 

15. There are still a few grammatical errors here and there.... please do a "thorough sweep-through" to ensure you clear them all...

Done. Proof-reading by native speaker was done.

Attachment Submitted filename: Response to editor.docx Click here for additional data file.

# Editor report: Decision Letter 2

**DOI:** 10.1371/journal.pone.0229916.r005

**Authors:** Yeetey Akpe Kwesi Enuameh

19 Feb 2020

Barriers and facilitators to contraceptive use among Somali immigrant women in Oslo: A qualitative study

PONE-D-19-23365R2

Dear Dr. Abdi A Gele,

We are pleased to inform you that your manuscript has been judged scientifically suitable for publication and will be formally accepted for publication once it complies with all outstanding technical requirements.

Within one week, you will receive an e-mail containing information on the amendments required prior to publication. When all required modifications have been addressed, you will receive a formal acceptance letter and your manuscript will proceed to our production department and be scheduled for publication.

Shortly after the formal acceptance letter is sent, an invoice for payment will follow. To ensure an efficient production and billing process, please log into Editorial Manager at https://www.editorialmanager.com/pone/, click the "Update My Information" link at the top of the page, and update your user information. If you have any billing related questions, please contact our Author Billing department directly at authorbilling@plos.org.

If your institution or institutions have a press office, please notify them about your upcoming paper to enable them to help maximize its impact. If they will be preparing press materials for this manuscript, you must inform our press team as soon as possible and no later than 48 hours after receiving the formal acceptance. Your manuscript will remain under strict press embargo until 2 pm Eastern Time on the date of publication. For more information, please contact onepress@plos.org.

With kind regards,

Yeetey Akpe Kwesi Enuameh, MD, MSc, DrPH

Academic Editor

PLOS ONE

Additional Editor Comments:

Admire the step by step approach to changes and reporting them also as such. Thank you.

Most of the issues have been addressed. The issues raised with the references are still outstanding though.

Page 11, line 5 would better read ".... mainly due to health system related factors"

Page 14, line 16 would better read "... women's utilization of contraceptives such as religious beliefs...."

Congratulations on the effort...

Reviewers' comments:

# Editor report: Acceptance letter

**DOI:** 10.1371/journal.pone.0229916.r006

**Authors:** Yeetey Akpe Kwesi Enuameh

24 Feb 2020

PONE-D-19-23365R2 

Barriers and facilitators to contraceptive use among Somali immigrant women in Oslo: A qualitative study 

Dear Dr. Gele:

I am pleased to inform you that your manuscript has been deemed suitable for publication in PLOS ONE. Congratulations! Your manuscript is now with our production department. 

If your institution or institutions have a press office, please notify them about your upcoming paper at this point, to enable them to help maximize its impact. If they will be preparing press materials for this manuscript, please inform our press team within the next 48 hours. Your manuscript will remain under strict press embargo until 2 pm Eastern Time on the date of publication. For more information please contact onepress@plos.org.

For any other questions or concerns, please email plosone@plos.org. 

Thank you for submitting your work to PLOS ONE.

With kind regards,

PLOS ONE Editorial Office Staff

on behalf of

Dr. Yeetey Akpe Kwesi Enuameh 

Academic Editor

PLOS ONE
